# Exploring risk factors for insect borer attack in Georgia’s (USA) urban landscapes

**DOI:** 10.1371/journal.pone.0299368

**Published:** 2024-02-26

**Authors:** Zia V. Williamson, Brett R. Blaauw, Shimat V. Joseph

**Affiliations:** 1 Department of Entomology, The University of Georgia, Griffin Campus, Griffin, GA, United States of Ameica; 2 Department of Entomology, The University of Georgia, Athens Campus, Athens, GA, United States of Ameica; USDA Forest Service Southern Research Station, UNITED STATES

## Abstract

Urban trees are at risk of stress due to heat island effects and the increased proportion of impervious areas surrounding them. Among pests of trees, insect borers such as bark beetles (Coleoptera: Curculionidae) and flatheaded borers (Coleoptera: Buprestidae) are some of the most devastating, frequently colonizing stressed trees. The objective of this study was to explore the effects of biotic and abiotic risk factors on borer attacks on trees in urban areas. In the summer of 2021 and 2022, this study was conducted in 50 urban sites in Atlanta and Augusta, Georgia (USA). Specific factors explored include overall tree health, differentially warmer maximum and minimum temperatures of sites compared to surrounding areas, tree species, and the percentage of impervious surface surrounding trees. Generalized linear models and zero-inflated models explored how these factors were related to damage from these borers. The number of borer attacks on trees increased with higher percentage impervious area. As the two most commonly encountered trees, *Acer rubrum* was found to be significantly more susceptible to attack from borers than *Ulmus parvifolia*. Unhealthy trees were more likely to experience more frequent and more severe borer attack. Trees with increased impervious cover around them as well as those with differentially warmer daily maximum and minimum temperatures relative to surrounding were more likely to be attacked.

## Introduction

Urban trees are an important part of our daily lives and landscapes. Trees can help improve the human perception of urban areas, providing visual and sensory benefits [1], besides increasing property value [2]. In addition, urban trees can provide vital ecosystem services such as carbon sequestration and air filtration, cooling through increased shade, reducing flooding resulting from storms, and providing habitat for urban animals [3]. As such, protecting urban trees through understanding the problems that they may encounter is essential.

Urban trees often experience stress at much higher rates than trees in surrounding natural areas, such as forests [4, 5]. In urban areas, proportion of impervious surfaces such as sidewalks, parking lots, and roads has dramatically increased [6]. These impervious surfaces reduce water infiltration and often have firmly packed and compacted soil beneath them, reducing the rootable soil volume available to trees [7]. In addition, because temperatures in urban areas increase as a result of higher absorption of solar radiation to those impervious surfaces, heat island effects may occur [5, 8]. These factors impose increased stress to trees. As a result of this increase in stress, trees in urban areas are more vulnerable to insect attacks than those trees in more forested areas [5, 9]. Other potential stressors include exposure to environmental pollutants and increased soil salt content due to de-icing procedures [10].

Planting trees in unfavorable urban sites, such as parking lots or right of ways may predispose trees to attack by insects or pathogens due to increased plant stress [10, 11]. It has previously been shown that numbers of gloomy scale, *Melanaspis tenebricosa* (Comstock), increased on urban trees as the percentage of impervious surface cover increased [5, 9]. Similarly, stressed trees have also been associated with increased attacks from many wood boring insects [12, 13]. However, the impact of stress factors on the relationship of urban trees with borer pests, such as flatheaded borers (Coleoptera: Buprestidae), ambrosia and bark beetles (Coleoptera: Curculionidae), and longhorned beetles (Coleoptera: Cerambycidae), is still not understood completely.

Flatheaded borers, such as the native flatheaded appletree borer *Chrysobothris femorata* (Olivier) and the invasive emerald ash borer, *Agrilus planipennis* Fairmaire, are widespread in much of the United States, impacting many host plant genera such as maples (*Acer*), oaks (*Quercus*), elms (*Ulmus*), redbuds (*Cercis*), and willows (*Salix*) [14–16]. The adults of flatheaded borers are bullet-shaped, and the larvae have a wide, flattened thoracic segment behind the head [15]. These borers cause damage primarily through the tunneling of larvae, especially into the trunk of the tree, which may lead to girdling of small trees over the years or the formation of cankers in some cases [15, 17–19]. The exit holes left by flatheaded borers have a characteristic “D” shape. Similarly, ambrosia beetles, such as *Xylosandrus crassiusculus* (Motschulsky) and *Xylosandrus germanus* (Blandford), and bark beetles, such as *Hypothenemus hampei* (Ferrari) and *Pityophthorus juglandis* (Blackman), are pests of deciduous tree species such as dogwoods (*Cornus)*, maples (*Acer*), oaks (*Quercus*), elms (*Ulmus*), redbuds (*Cercis*), and walnut *(Juglans)* [19, 20]. Ambrosia beetles primarily attack the tree trunk, bore through vascular bundles, and construct fungus-inoculated galleries in the heartwood, while bark beetles attack and feed on the inner bark [19, 20]. Entry holes left by ambrosia and bark beetles (hereafter, more generally referred to as bark beetles) are approximately 1 mm in diameter [17]. Longhorned beetles, such as prionids and *Monochamus* spp. [21–23], are common in Georgia (USA), and can potentially attack trees in urban landscapes. The invasive Asian longhorned beetle, *Anoplohora glabripennis* Motschulsky, poses a threat to trees [22], although it is not reported from Georgia. Longhorned beetle larval boring causes splitting of the tree bark, and the exit holes of the adult longhorned beetle are large and round-shaped [22, 24]. Hosts vary between species, with some groups feeding upon on dead wood and others in live tree tissues [21].

As developers and planners of urban locales continue to emphasize and implement urban forestry through these areas, practices such as increasing green spaces and utilizing sustainable management of pests such as tree-boring beetles will become increasingly necessary [25]. Despite the direct impact of these pests, little work has been conducted to explore risk factors that enhance borer attacks in urban settings, especially in Georgia. Because of this, understanding the various factors that may place trees at higher risk of borer attacks is essential. By exploring ways to prevent or mitigate attacks in the first place, the overall longevity of trees can be improved, and economic loss associated with the death of trees as a result of insect borers can be reduced. As such, the objectives of our study were to determine 1) the proportion of trees in urban sites damaged due to borer activity and 2) the influence of biotic and abiotic characteristics contributing to borer infestations in urban landscapes.

## Materials and methods

### Study sites

In 2021 and 2022, a study was conducted in urban landscapes of Atlanta and Augusta, Georgia (USA). In 2021, 887 trees were selected from 30 urban sites for sampling, whereas in 2022, 474 trees were selected from 20 new sites for a total of 1351 trees (S1 Table). Google Earth Pro version 7.3.6 was used for site selection utilizing the time progression feature. Most of the selected sites had trees planted within the last 15 years; however, five and three sites in 2021 and 2022, respectively, had trees predating that period. The types of sites included in the study ranged from subsections of large shopping centers to smaller green spaces, such as public parks and their associated parking lots. Each site was visited once from early June to early August. More details about selected sites are provided in S1 Table.

### Biotic factors

Both biotic and abiotic factors were measured with the response variables of flatheaded borer, longhorned beetle, and bark beetle damage. Borer damage was quantified by counting visible entry or exit holes on the tree trunk at a height of ≤ 1.5 m from the ground. Single-trunked deciduous trees were evaluated. As entry holes of flatheaded borers often occur through wounds and are cryptic, the exit holes utilized in the study provide clear evidence of larval development and subsequent adult emergence. D-shaped exit holes and round pinholes were quantified as signs of flatheaded borer or bark beetle emergence [17, 19, 24].

Overall tree health was assessed and assigned a value ranging from 1 to 4 using a scale system similar to that described by Dale et al. [9]. Tree health was rated as dead, poor, fair, or good, with values of 1 to 4, respectively. Trees were rated dead when devoid of leaves, but with brittle twigs. Trees rated as poor had multiple dead branches, severely damaged central leaders, or a majority of the canopy was dead. Trees rated as fair condition had one dead branch or intermediate canopy dieback. Trees in good condition had minimal damage (cankers, scrapes, self-girdling, and leaf scorch), branch breakage, or canopy dieback. These injury symptoms can be manifested because of tree stress or disease incidence in addition to borer related activity. Thus, tree health rating could be partially confounded with other unknown factors. The species of trees was determined, and the trunk diameter was measured using a Vernier caliper at breast height or at the point where the first branch forked, whichever was the shortest distance from the ground.

### Abiotic factors

**Fig 1:**
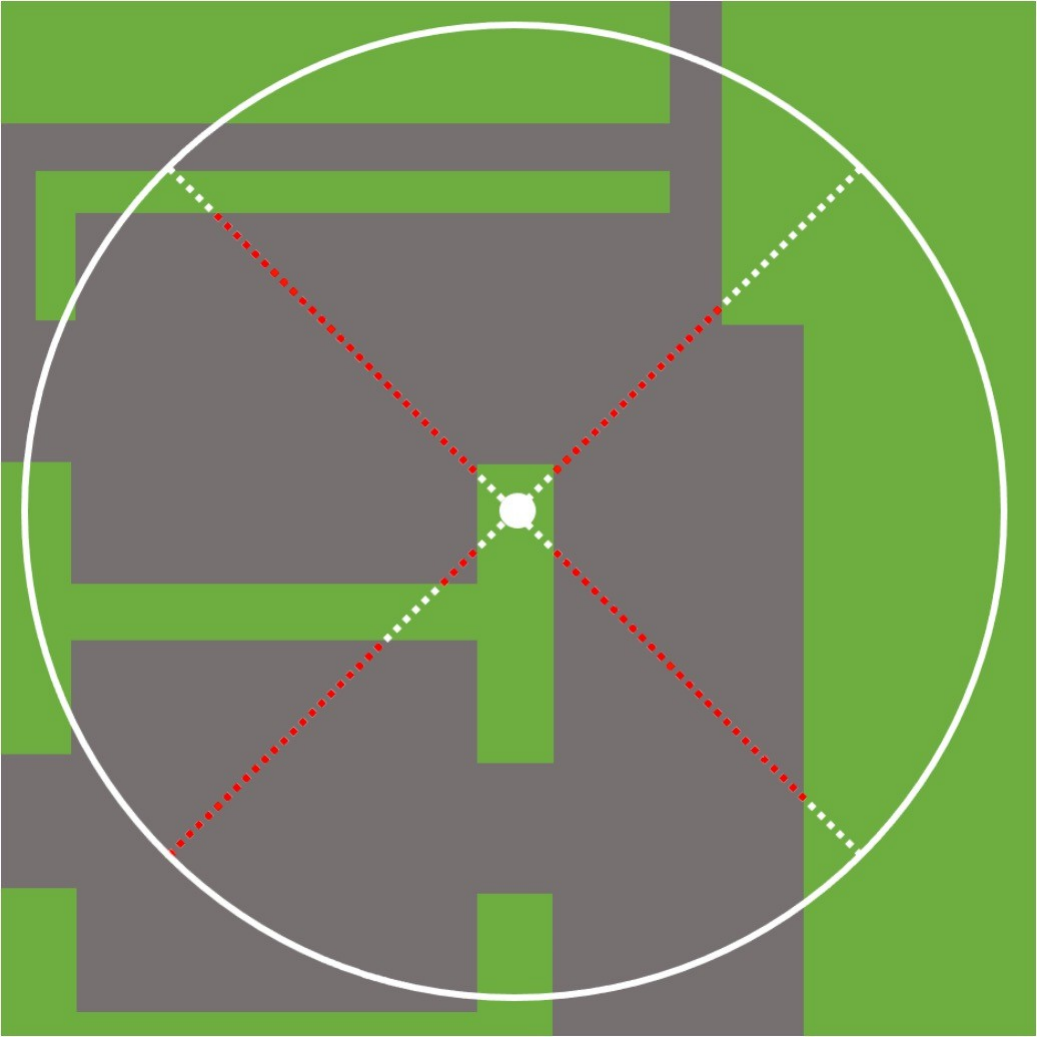
Graphic demonstration of the Pace-to-Plant technique as used in Dale et al. [5]. Four transects were marked with dashed lines originating at the tree base. Transects were at 90°, with the first transect positioned at 45° to the closest impervious edge, such as pavement or sidewalk. Each transect consisted of 25 steps, with 100 steps in total. The number of steps on the impervious surface, represented by the red portion of the line, out of those 100 were recorded.

To determine the relationship between abiotic factors and incidence of borer damage the percentage of impervious surface surrounding trees and air temperature were recorded from all study sites. The percentage of impervious surface was estimated using the “Pace-to-Plant” technique described by Dale et al. [5]. Starting from the tree, the person moved 25 steps in four transects at 90° in a “X” pattern. The “starting” transect was determined at 45° angle to the closest impervious edge (Fig 1). The number of steps on a pervious surface and an impervious surface were recorded. The estimated percentage of the impervious area surrounding the tree was calculated by dividing the number of steps on the impervious surface by the total number of steps taken in each transect. This technique was administered by a single person throughout the study to maintain consistency. These measurements were documented for every tree at a given site.

One temperature logger was deployed in a subsection of randomly selected sites. Loggers (HOBO, Bourne, Massachusetts, USA; Part# UA-002-08) were placed at 2.1 m above ground to reduce public interference with the loggers. The temperature loggers were protected by placing them into a transparent plastic cup. In 2021, 13 loggers were deployed on 1 July and were recovered from the urban sites on 3 August. In 2022, 12 loggers were placed in various sites and deployed on 1 July and retrieved on 3 August. Temperature data was recorded every hour for the duration of the study The daily maximum and minimum temperature data were retrieved from the logger devices using HOBOware software (Onset Computer Corporation, Bourne, Massachusetts).

Local maximum and minimum temperatures were obtained from the nearest University of Georgia Weather Network station for each site, and the difference between the daily maximum and minimum temperature of the logger and the daily maximum and minimum temperature of the weather station was determined. In all cases, the temperature in sites was warmer than that gathered from the weather stations in the surrounding area. The daily differences between the loggers and stations were then averaged to create an average deviation of how much warmer maximum and minimum temperatures for each site were than those in the surrounding areas, further referred to as differential temperature increase. One and two loggers’ data were lost due to tampering or removal of branches by landscape professionals in 2021 and 2022, respectively.

### Statistical analyses

All statistical analyses were conducted utilizing R software version 4.1.3 [26]. The data obtained were not normally distributed when the response variables of flatheaded borer damage and bark beetle damage were tested. Longhorned beetles were excluded from all analyses due to limited incidence.

Because no attack was observed on many trees, a generalized linear model following the zero-inflated negative binomial distribution was considered, as in earlier published papers [27, 28]. Zero-inflated models, specifically zero-inflated negative binomial models, are suited for exploring relatively infrequently encountered species that tend to aggregate when present for not well-known reasons. As described in Minami [27], zero-inflated models are divided into two distinct states: 1) An imperfect state, where, in the case of current study, incidence of attack was possible, and 2) a perfect state, where no incidences of attack are expected. Zero-inflated negative binomial models have two distinct phases: The perfect, or zero-inflated, phase of the model follows the binomial distribution and can be interpreted as a positive coefficient reflecting an increased likelihood of being in the “perfect” or non-attacked group. The alternative is the “possible attack” group which makes up the “imperfect” phase of the model. The imperfect phase functions as a typical count model, and displays what factors contribute to the severity of borer attacks when they do occur to a given tree [29].

To conduct the analysis, the “pscl” package was implemented through the use of the “zeroinfl()” function to construct the zero-inflated negative binomial models [30, 31]. Candidate models initially included all abiotic factors, tree diameter, and health rating as stand-alone variables. Models were constructed and selected for overall suitability based on backward stepwise regression, with comparisons of Akaike information criterion scores and R-squared values of all candidate models. The Vuong test determined whether the zero-inflated model provided the best fit, with the zero-inflated negative binomial model proving to be significantly better than the negative binomial model for both borer damage types. Nonlinear exponential regression was also explored as an option for statistical analysis. Exponential regression models were compared to the zero-inflated negative binomial models on the basis of Akaike information criterion scores and Bayesian information criterion scores. In the case of all three borer classification models, the zero-inflated negative binomial models had lower scores, indicating better relative fit. These scores are presented in S2 Table.

All processes previously described were repeated with each insect grouping, e.g., flatheaded borer damage or bark beetle damage serving as the response variable, as well as with bark beetles and flatheaded borer damage types combined for “borers in general.” Data from the 2021 and 2022 survey seasons were combined due to insignificant year effect when used as the sole parameter in a zero-inflated negative binomial model of all damage types evaluated.

Based on the Akaike information criterion scores and R-squared values of candidate models, the selected zero-inflated negative binomial models for incidence of damage from flatheaded borer, bark beetle, and both borer types together included the following factors: Tree health rating, percentage impervious area, and differentially warmer maximum and minimum temperatures. Post-hoc multiple comparisons of estimate marginal means of borer holes as influenced by tree health rating were conducted using the “emmeans” package of R to complete EMMEANS testing using the Tukey P value adjustment method.

The susceptibility of tree species was analyzed independently using a generalized linear model following the negative binomial distribution. Any tree species with < 13 replications were excluded from the analysis based on equivalency of < 1% of the overall population of trees sampled, which was 13 tree species. Post-hoc multiple comparisons of estimated marginal means of damage to tree species were conducted using the “emmeans” package of R to complete EMMEANS testing using the Tukey P value adjustment method. In the case of our study, degrees of freedom were considered to be infinite as a result of asymptotic results obtained from the use of the z-statistic in the negative binomial model.

Distributions of percent impervious area and temperature are included in S1 Fig.

## Results

We surveyed 1351 trees in the study in 2021 and 2022. Of 1351 trees surveyed in 2021 and 2022, 8.8% had flatheaded borer attacks, with 1001 exit holes in total. Similarly, 2.7% of trees had bark beetles entry holes, with 438 holes in total. Three trees had longhorned beetle entry holes, with 14 total holes. Only 0.67% of trees were attacked by both flatheaded borers and bark beetles.

**Fig 2:**
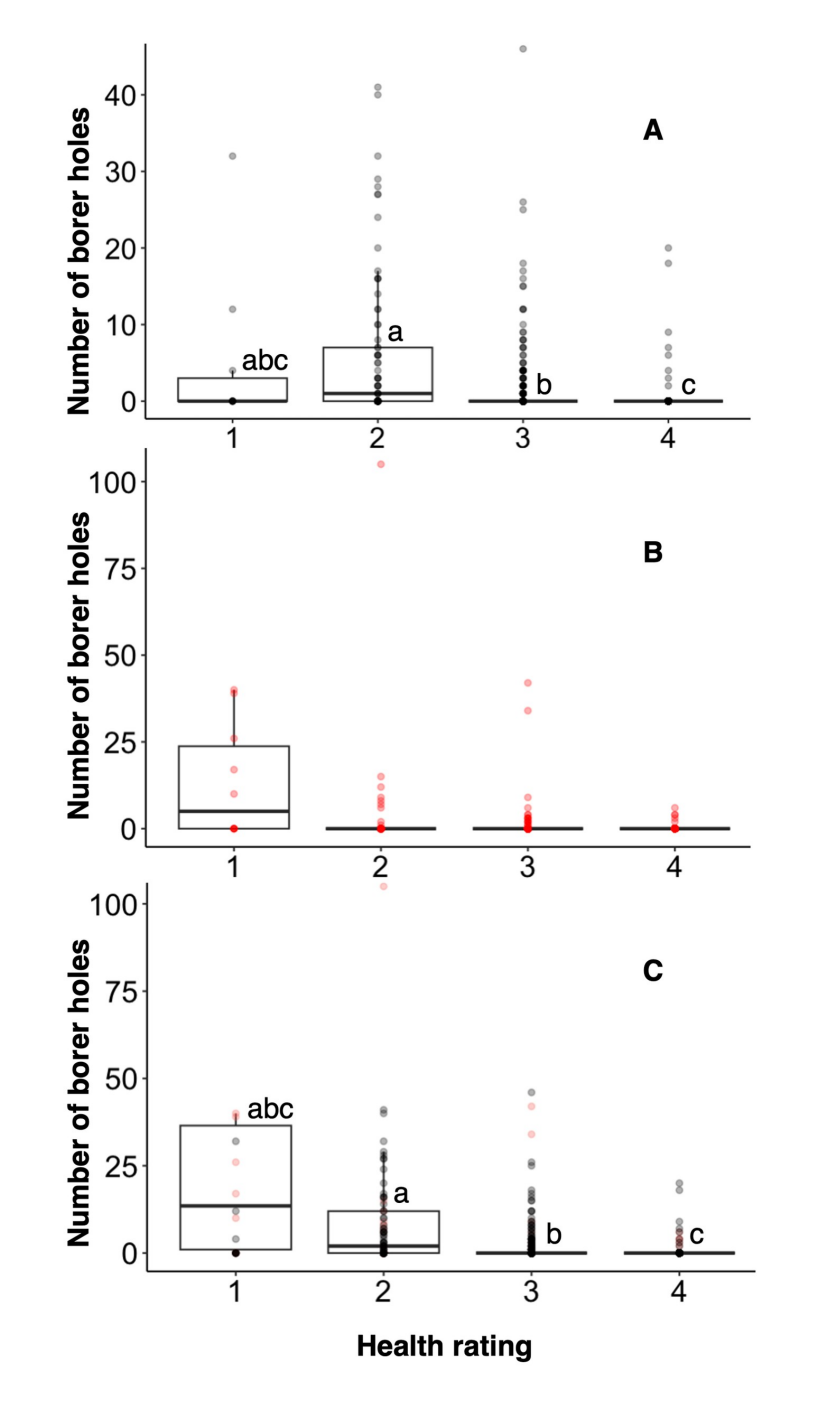
The number of borer holes by health rating. A health scale was used where 1, dead; 2, poor health; 3, fair health; and 4, good health for (A) flatheaded borer, (B) bark beetle damage, and (C) overall borer damage. Flatheaded borer attacks are black in color, and bark beetle attacks are red in color. Boxes with the same letters are not significantly different (EMMEANS, α = 0.05).

A total of 734, 530, 78, and 10 trees were rated as good, fair, poor and dead, respectively. Tree health rating values were analyzed using the zero-inflated negative binomial model with post-hoc EMMEANS testing (Table 1). In terms of flatheaded borer and overall borer damage, “poor” or “fair” rated trees were significantly different from “healthy” trees, with healthy trees having fewer borer holes. “Fair” trees were significantly different from “healthy” and “poor” trees, having more borer holes than “healthy” trees, but fewer than “poor” trees (Table 2, Fig 2A and 2C), There were no significant differences between health ratings for bark beetle damage (Table 2 and Fig 2B).

**Table 1:** Coefficients of parameters by borer type for the count model portion and the zero-inflation portion of the zero-inflated negative binomial models for sites visited in 2021 (n = 30) and 2022 (n = 20). Tree health was rated as dead, poor, fair, or good, with values of 1–4, respectively.

Factor	Count Model Portion					Zero Inflated Portion				
	Estimate	SE	Z	*P*	Effects	Estimate	SE	Z	*P*	Effects
**Flatheaded borer**										
Intercept	-2.000	1.570	-1.274	0.203		1.086	1.744	0.623	0.534	
Tree health rating of 1	0.120	0.911	0.132	0.895		-4.140	1.135	-3.648	**< 0.001**	**-**
Tree health rating of 2	0.328	0.719	0.456	0.649		-5.561	0.793	-7.013	**< 0.001**	**-**
Tree health rating of 3	-0.504	0.663	-0.761	0.447		-3.249	0.615	-5.279	**< 0.001**	**-**
Percent impervious area	-0.029	0.010	-2.944	**0.003**	-	0.057	0.016	3.662	**< 0.001**	**+**
Mean deviation of max temperature	0.210	0.141	1.494	0.135		-0.098	0.142	-0.693	0.488	
Mean deviation of min temperature	0.468	0.194	2.409	**0.016**	**+**	-0.376	0.249	-1.509	0.131	
**Bark beetle**										
Intercept	-2.361	4.233	-0.558	0.577		5.281	2.169	2.435	0.015	**+**
Tree health rating of 1	2.490	0.684	3.638	**< 0.001**	**+**	-4.599	1.021	-4.506	**< 0.001**	**-**
Tree health rating of 2	1.532	0.702	2.183	**0.029**	+	-2.064	0.856	-2.409	**0.016**	**-**
Tree health rating of 3	1.250	0.618	2.021	**0.043**	+	-0.877	0.674	-1.301	**0.193**	
Percent impervious area	0.007	0.032	-0.228	0.820		0.000	0.018	0.017	0.987	
Mean deviation of max temperature	0.528	0.295	1.790	0.074		-0.438	0.207	-2.119	**0.034**	-
Mean deviation of min temperature	-0.139	0.491	-0.282	0.778		0.759	0.537	1.414	0.157	
**Borers overall**										
Intercept	-3.215	1.349	-2.383	**0.017**	**-**	0.611	1.698	0.360	0.719	
Tree health rating of 1	1.369	0.613	2.233	**0.026**	**+**	-17.998	487.687	-0.037	0.971	
Tree health rating of 2	1.103	0.529	2.087	**0.037**	**+**	-4.756	0.617	-7.705	**< 0.001**	**-**
Tree health rating of 3	0.166	0.492	0.337	0.736		-2.508	0.4601	-5.451	**< 0.001**	**-**
Percent impervious area	-0.031	0.009	3.643	**< 0.001**	-	0.052	0.015	3.577	**< 0.001**	**+**
Mean deviation of max temperature	0.358	0.121	2.961	**0.003**	+	-0.143	0.129	-1.108	0.268	
Mean deviation of min temperature	0.379	0.167	2.272	**0.023**	+	-0.230	0.233	-0.985	0.325	

Black plus sign indicators [+] denote a positive effect. Grey minus sign indicators [–] denote a negative effect.

**Table 2:** Summary of the post-hoc estimated marginal means analysis for the effect of tree health rating on number of borer holes. Groups with the same letter did not differ statistically (*p*>0.05*)*.

Tree Rating	emmean	SE	df	asymp.LCL	asymp.UCL	Group
**Flatheaded borers**						
**1**	3.0928	2.6374	Inf	-2.0763	8.262	abc
**2**	7.0852	1.9351	Inf	3.2925	10.878	c
**3**	0.8818	0.2105	Inf	0.4693	1.294	b
**4**	0.0708	0.0545	Inf	-0.0359	0.178	a
**Bark beetles**						
**1**	9.4993	5.0758	Inf	-0.44913	19.4477	a
**2**	0.5656	0.4495	Inf	-0.31534	1.4466	a
**3**	0.1382	0.0739	Inf	-0.00664	0.2829	a
**4**	0.0167	0.0115	Inf	-0.00588	0.0393	a
**Borers overall**						
**1**	16.3753	6.374	Inf	3.8822	28.87	abc
**2**	9.2411	2.235	Inf	4.8603	13.62	c
**3**	1.1188	0.233	Inf	0.6626	1.58	b
**4**	0.0976	0.052	Inf	-0.0043	0.20	a

**Fig 3:**
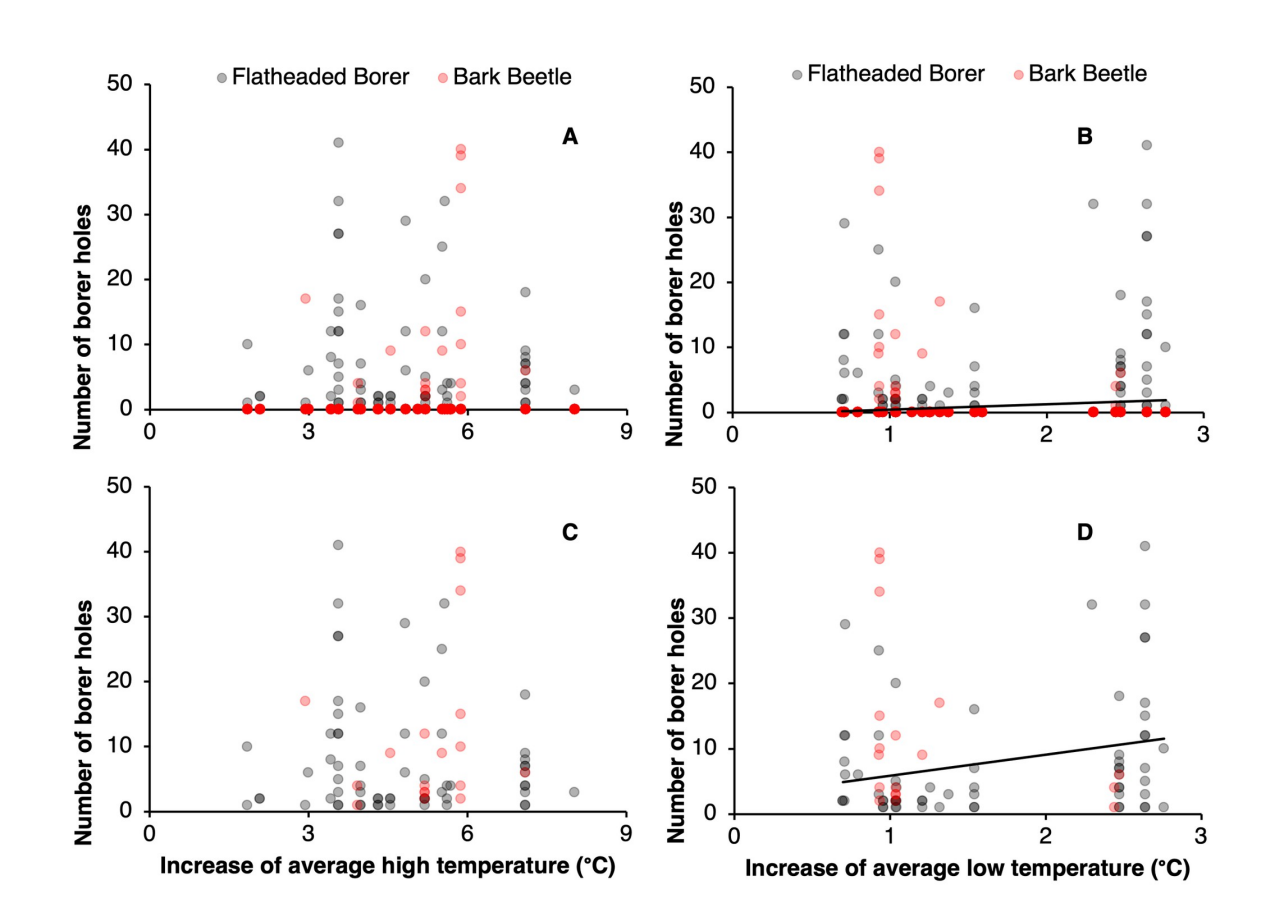
The numbers of holes of flatheaded borer and bark beetles by differentially warmer temperatures. Holes were recorded on the tree in relation to the average increase of ambient air temperature of each site compared to the surrounding area’s average maximum (A, C) and (B, D) minimum temperatures. Plots including (A, C) and excluding (B, D) trees without borer holes are included. Temperature loggers were placed in experimental sites from 1 July to 3 August in 2021 and 2022.

A significantly greater number of flatheaded borer exit holes can be expected when the low temperatures of an area are differentially higher than those of the surrounding area (Table 1 and Fig 3A). Although no temperature-related parameter had a significant effect on bark beetle damage counts, trees in sites where the maximum temperature of the area was differentially higher than the surrounding area were more likely to be in the “no attack” group due to that parameter being significant with a positive estimate (Table 1 and Fig 3B). However, the current study did not explain the exact variable (s) triggering those attacks. When all the damage types were combined, the increased differentially warmer maximum and minimum temperature of a site contributed to increased borer attack (Table 1 and Fig 3C).

**Fig 4:**
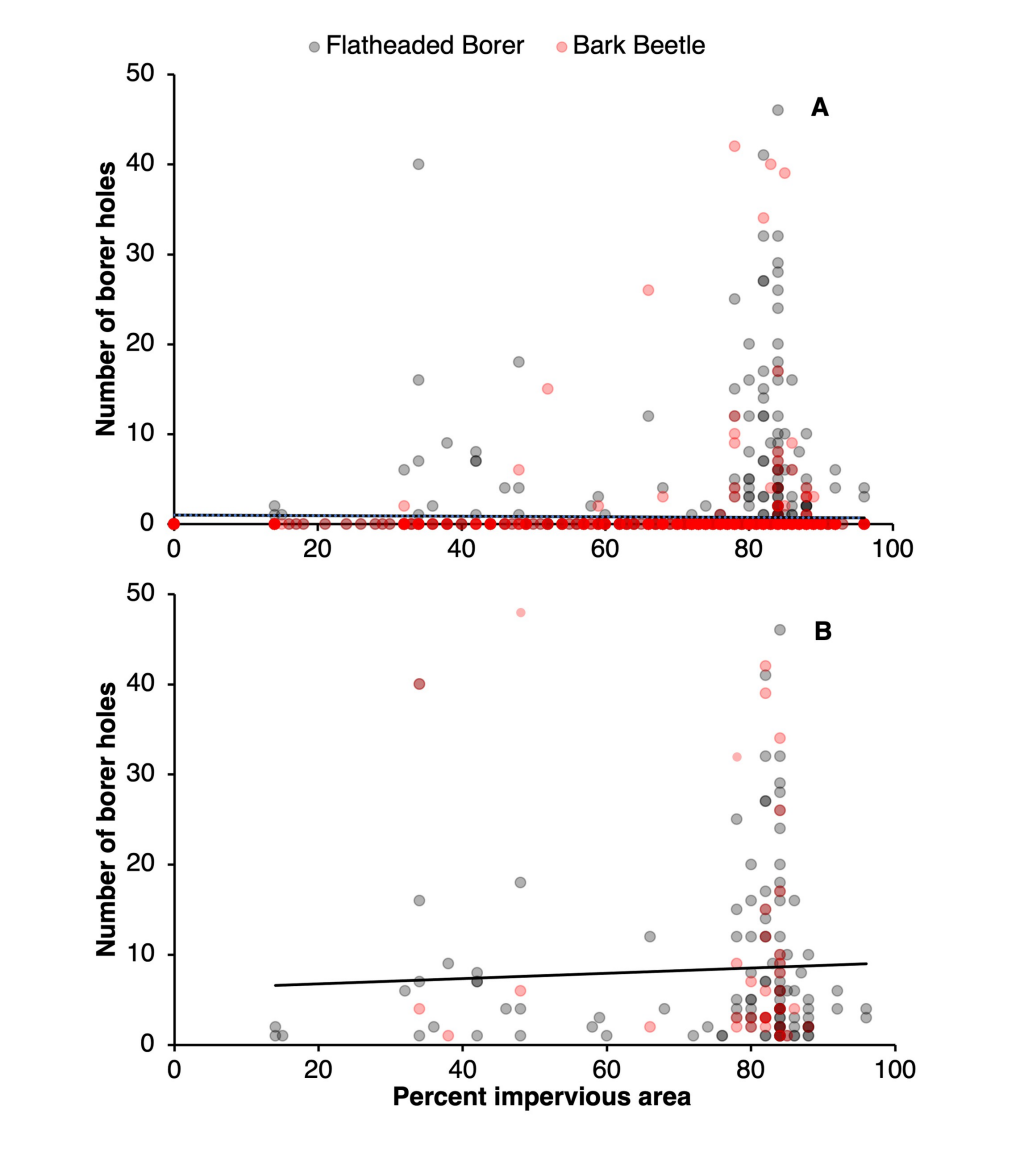
The numbers of holes of flatheaded borer and bark beetles by the percentage of pervious area. Holes were recorded on trees in addition to the percentage of pervious area around each tree where (A) zero incidences of attack were included and (B) excluded. The percentage of impervious area was determined using the “Pace-to-Plant” technique.

The damage from flatheaded borers and all borers combined significantly increased with an increase in the percentage of impervious cover. In contrast, there was no significant relationship between bark beetle damage and the percentage of impervious cover (Table 1, Fig 4A and 4B). However, based on the count model, an increased percentage of impervious cover was related to more severe damage from flatheaded borers and all borers combined; the zero-inflation portion of the models demonstrated that with increasing pervious cover, the less likely trees are to be in the “no attack” group from flatheaded borers and all borers combined (Table 1, Fig 4A and 4B).

**Fig 5:**
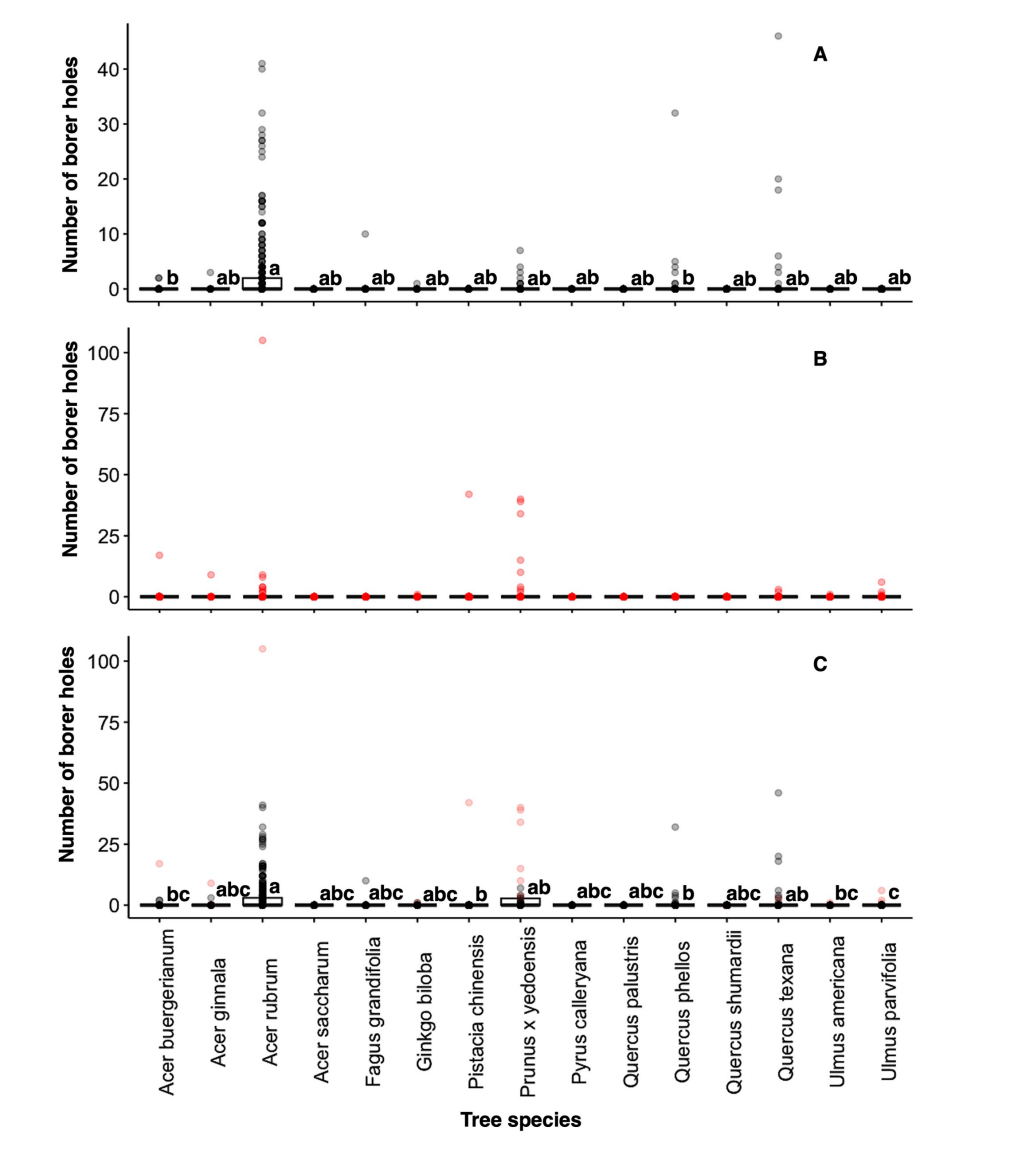
The numbers of holes per tree of (A) flatheaded borer, (B) bark beetles, and (C) all borers combined by tree species. Flatheaded borer attacks are in black, and bark beetle attacks are in red. Boxes with the same letters are not significantly different (EMMEANS, α = 0.05).

Among tree species, the number of flatheaded borer attacks were higher for red maple, *A*. *rubrum*, which had significantly more attacks than *A*. *buergerianum* and *Q*. *phellos* (Table 3 and Fig 5A). There were no significant differences between tree species for number of bark beetle holes (Table 3 and Fig 5B). *A*. *rubrum* experienced significantly more incidences of borer attack in general than *Ulmus parvifolia*, with those trees belonging to the group with the highest and lowest number of attacks, respectively (Table 3 and Fig 5C). More detailed results of other comparisons are presented in Table 3. The number of holes in trees from a given borer type are given in S3 Table.

**Table 3:** Summary of the post-hoc estimated marginal means analysis for the effect of tree species on number of borer holes. Groups with the same letter did not differ statistically (*p*>0.05*)*. Results are given on the log scale.

Tree Rating	n	emmean	SE	df	asymp.LCL	asymp.UCL	Group
**Flatheaded borers**							
*Acer buergerianum*	20	-2.674	0.715	Inf	-4.080	-1.272	a
*Acer ginnala*	14	-1.540	1.191	Inf	-3.880	0.794	ab
*Acer rubrum*	240	1.085	0.254	Inf	0.586	1.583	b
*Acer saccharum*	32	-22.303	7468.314	Inf	-14700.000	14615.325	ab
*Fagus grandifolia*	26	-0.956	0.827	Inf	-2.580	0.666	ab
*Ginkgo biloba*	18	-2.890	1.358	Inf	-5.550	-0.229	ab
*Pistacia chinensis*	103	-22.303	4162.737	Inf	-8180.000	8136.512	ab
*Prunus* x *yedoensis*	37	-0.693	0.673	Inf	-2.010	0.625	ab
*Pyrus calleryana*	20	-22.303	9446.753	Inf	-18500.000	18492.994	ab
*Quercus palustris*	34	-22.303	7245.329	Inf	-14200.000	14178.282	ab
*Quercus phellos*	128	-1.023	0.375	Inf	-1.760	-0.289	a
*Quercus shumardii*	81	-22.303	4694.130	Inf	-9220.000	9178.022	ab
*Quercus texana*	135	-0.320	0.350	Inf	-1.010	0.366	ab
*Ulmus americana*	26	-22.303	8285.351	Inf	-16300.000	16216.687	ab
*Ulmus parvifolia*	274	-22.303	2552.245	Inf	-5020.000	4980.005	ab
**Bark beetles**							
*Acer buergerianum*	20	-1.227	1.286	Inf	-3.75	1.294	a
*Acer ginnala*	14	-0.442	2.593	Inf	-5.52	4.64	a
*Acer rubrum*	240	-0.561	0.627	Inf	-1.79	0.668	a
*Acer saccharum*	32	-22.303	7468.315	Inf	-14659.93	14615.325	a
*Fagus grandifolia*	26	-22.303	8285.351	Inf	-16261.29	16216.687	a
*Ginkgo biloba*	18	-2.890	2.479	Inf	-7.75	1.968	a
*Pistacia chinensis*	103	-0.897	0.961	Inf	-2.78	0.986	a
*Prunus* x *yedoensis*	37	1.353	1.563	Inf	-1.71	4.416	a
*Pyrus calleryana*	20	-22.303	9446.754	Inf	-18537.6	18492.994	a
*Quercus palustris*	34	-22.303	7245.329	Inf	-14222.89	14178.282	a
*Quercus phellos*	128	-22.303	3734.157	Inf	-7341.12	7296.511	a
*Quercus shumardii*	81	-22.303	4694.13	Inf	-9222.63	9178.022	a
*Quercus texana*	135	-3.296	0.941	Inf	-5.14	-1.451	a
*Ulmus americana*	26	-3.258	2.136	Inf	-7.44	0.928	a
*Ulmus parvifolia*	274	-3.416	0.67	Inf	-4.73	-2.103	a
**Borers overall**							
*Acer buergerianum*	20	-1.016	0.6	Inf	-2.192	0.16	ab
*Acer ginnala*	14	-0.154	1.173	Inf	-2.454	2.145	abc
*Acer rubrum*	240	1.261	0.277	Inf	0.719	1.804	c
*Acer saccharum*	32	-20.303	2747.439	Inf	-5405.185	5364.58	abc
*Fagus grandifolia*	26	-0.956	0.892	Inf	-2.705	0.794	abc
*Ginkgo biloba*	18	-2.197	1.227	Inf	-4.602	0.208	abc
*Pistacia chinensis*	103	-0.897	0.447	Inf	-1.773	-0.021	b
*Prunus* x *yedoensis*	37	1.474	0.695	Inf	0.113	2.836	bc
*Pyrus calleryana*	20	-20.303	3475.267	Inf	-6831.7	6791.095	abc
*Quercus palustris*	34	-20.303	2665.408	Inf	-5244.406	5203.801	abc
*Quercus phellos*	128	-1.023	0.404	Inf	-1.815	-0.232	b
*Quercus shumardii*	81	-20.303	1726.874	Inf	-3404.913	3364.308	abc
*Quercus texana*	135	-0.271	0.379	Inf	-1.014	0.473	bc
*Ulmus americana*	26	-3.258	1.302	Inf	-5.811	-0.705	ab
*Ulmus parvifolia*	274	-3.416	0.421	Inf	-4.241	-2.591	a

## Discussion

The severity of attacks from flatheaded borers and borers overall increased with a higher proportion of impervious surface surrounding a tree. However, a recent study shows that tree cover in urban areas of the USA is declining and overall impervious cover is increasing [32]. This suggest that urban trees could be more likely to be exposed to stressors such as intermittent flooding or drought events [33, 34]. The construction and development of urban areas and subsequent increased soil compaction can affect the water and nutrient uptake of trees and contribute to increased stress [35]. As stressed trees are known to attract flatheaded borers, more severe attack can be associated with increased impervious area [12, 33, 36]. Although an increase in impervious area was associated with more severe attacks from both flatheaded borers and borers in general, the zero-inflated model indicated that trees in areas with lower impervious surfaces also experienced a higher likelihood of attack. Although the exact reasons for this contrasting result are unclear, one possibility could be related to high counts of flatheaded borers in surrounding forested areas. While forest cover adjacent to sites was outside the scope of the current study and was not quantified, more research is warranted with a specific focus on the impact of factors related to forest cover adjacent to urban sites. This paradox highlights a need for further research. As this study is based on observation of dynamic areas, exploring this phenomenon in other locales may provide more insight into the potential effects of impervious surface cover on flatheaded borers in urban areas.

With the continuous growth of cities, increasing amounts of urban sprawl, and growing concern for heat island effects, the correlation between insect borer attacks and warmer temperatures of the sites surveyed is troubling [5, 37–39]. Results show that flatheaded borer attacks and the severity of the attack increased with differentially warmer low temperatures. In contrast, bark beetle attacks became more likely with differentially warmer maximum temperatures than that of surrounding areas as per the zero-inflation model. Previously, *A*. *planipennis*, an invasive flatheaded borer destroying ash trees in the USA, has been shown to have expedited rates of larval development in warmer climates in China [16]. These past studies and results from the current study suggest that flatheaded borer damage in urban areas could be, at least partially, influenced by warmer temperatures in urban areas [13, 16, 40]. Going forward, understanding how temperatures specifically influence flatheaded borer development is necessary and may aid in developing management and development guidelines to mitigate damage from these insects.

Trees in urban areas with differentially warmer maximum temperatures than surrounding areas were more prone to bark beetle attacks based on the zero-inflation model, although they were not likely to have more severe attacks if maximum temperatures increased based on the count model portion. This result poses unique questions regarding the presence of these insects in urban areas, with motivation for initial attack and continuing attack appearing to have different drivers. Previously, Bellahirech et al. [39] showed a correlation between increasing average temperatures and increased colonization of bark beetles in cork oak, *Quercus suber*. Nevertheless, another study showed that an increase in temperatures did not correspond to higher abundance of Kuroshio shot hole borer, *Euwallacea Kuroshio* Gomez and Hulcr 2018, emerging and as temperatures became warmer, emergence eventually ceased [41]. Thus, beetle reproduction and the emergence of bark beetles in urban areas could not be merely explained by changes in maximum temperatures alone. This highlights the need for future studies, with the methodology for this paper likely being hindered by the removal of severely impacted trees. Further studies may revolve around the effects of temperature on attack, emergence, and colonization in more controlled settings where tree material is not removed.

Urban warming can induce heat-related stress in trees, leading to declines in overall tree health. These stressors can play a role in the occurrence and severity of borer attacks, especially flatheaded borers, in urban areas. Exotic bark beetles are known to colonize stressed or freshly dead trees [14, 42–44]. In our study, bark beetles displayed no significant preference in terms of health of tree hosts, with dead, poor health, fair health, and healthy trees all being attacked similarly. In comparison to bark beetles, flatheaded borers were significantly more likely to attack stressed, but not dead, trees in comparison to healthy trees. This may result from the feeding habits of flatheaded borers, which tunnel through the tree and consume living tree tissue [16, 40]. This is consistent with previous findings, as flatheaded borers feed on stressed or dying, yet still alive, tissues in plants [45, 46]. In the study, dead trees were represented less frequently than other trees considered to be in poor, fair, or good health. These dead trees may have been removed as a result of poor aesthetics, skewing the data. This is of particular interest when considering bark beetles that have been recorded as occupying dead trees, potentially causing those species to be undersurveyed though visual inspection damage in urban areas [43, 44]. Thus, further understanding of these insects in urban areas may be reliant on other sampling techniques that conserve dead tree material in these areas.

Both flatheaded borers and bark beetles colonize various host trees [14, 47, 48]. In the current study, certain trees were found differentially susceptible to borer attacks in urban areas. Based on the common trees observed in the urban locations surveyed, *A*. *rubrum* was widely susceptible to borers in urban landscapes. As an understory tree, *A*. *rubrum* grows quickly in a landscape and thrives in various environmental conditions [49–51]. However, the quick growth of *A*. *rubrum* makes it susceptible to physiological defects related to internal structural issues and mechanical injury. These issues ultimately weaken the tree and make it more susceptible to attack from insect borers [50, 52]. In the USA, *A*. *rubrum* accounts for 10% of all deciduous shade trees sold and is estimated at ~$11 million USD per year [53]. Thus, their high susceptibility to borers can have major economic implications. The stressed trees then release volatiles, making them an attractive host to tree-boring beetles [1, 54]. This information suggests that stakeholders should carefully consider the plants utilized in landscapes, with care taken to select trees that are well suited to urban environments and resistant to the pests that occur in those areas.

In contrast to *A*. *rubrum*, *U*. *parvifolia* is resistant to borer attack in the current study. *Ulmus parvifolia* has developed a reputation for sturdy street trees for the conditions in the southeastern USA [55]. These results are consistent with previous studies, as *U*.*parvifolia* is recognized as one of the most pest-resistant trees in the genus *Ulmus* [56]. In addition, *U*. *parvifolia* tolerates frequent droughts and is suited for urban landscapes [55]. As young trees may be killed in one season after being attacked by flatheaded borers, urban planners and landscape professionals should consider planting more resistant tree species, such as *U*. *parvifolia*, that are less prone to borer attack to reduce the cost and burden on the customer in terms of long term implications [13, 15].

This study presented unique limitations inherent to urban spaces, crucial for data interpretation. Firstly, although trees were identified to species level, we could not identify specific cultivars. Susceptibility to pest attack and a tree’s response to infestation could vary among cultivars of a tree species [54, 56]. The cultivar information for specific tree species was not accessible to property owners or managers as that information was not recorded. The survey was limited to trees that remained in the landscape. As cankers and declines in tree health resulting from insect borer colonization are detrimental to the overall appearance of the landscape, afflicted trees may have been removed, causing those trees to be omitted from the study [18, 52]. Finally, borer damage was quantified solely on the trunk of the tree. Most economically important tree-boring pests attack the trunk of trees, with a minority of other species potentially attacking twigs and small branches in the canopy [46].

Trees in urban landscapes and the people that manage them face unique challenges, with concerns from the public and limitations on effective intervention methods [57, 58]. Despite this, urban trees are essential in mitigating some environmental concerns associated with urban areas [37, 38, 44, 58, 59]. Factors such as the percentage of impervious area, tree species, and the urban heat island phenomenon can play a role in influencing borer attacks on urban trees. Landscape planners and policymakers should factor in strategies to reduce impervious areas and dampen temperature surges in urban settings when developing urban and suburban areas to mitigate damage and subsequent economic loss and ecological disruption of urban trees [1, 3, 60]. Consequently, landscape managers, arborists, and landowners should utilize trees species suitable for urban areas to prevent economic loss. While management is often highly site specific, utilizing tree species that are less susceptible to insect borer attack may reduce losses, ultimately increasing success of trees in the area. In economic losses and loss of function in those areas may be mitigated. Furthermore, preventing stress to trees through implementation of tree care like proper pruning and irrigation may also help in reducing impacts from borers.

This research has wide-reaching implications regarding the impact of heat island effects and urbanization on the health of trees and our quality of life. Future research warrants increasing our understanding of how temperature influences colonization and the emergence of flatheaded borers and bark beetles and exploring both tree species and cultivar-related differences in insect borer vulnerability. In addition, understanding how the nature, health, and biodiversity of forest cover near urban areas influence the ecology, borer activity, and damage is of interest.

## Supporting information

S1 Table Details of the location and characteristics of selected sites in urban areas in Atlanta, GA (A) and Augusta, GA (B) were surveyed from July to August 2021 and 2022.
(DOCX)

S2 Table AIC and BIC scores for the comparison of nonlinear exponential regression and zero inflated negative binomial models. (DOCX)

S3 Table Number of flatheaded borer (A), bark beetle (B), and total borer (C) holes by tree species.
(DOCX)

S1 Fig The distributions of the independent variables. Distributions of percent impervious area (A), increase of average higher temperature (B), and increase of average low temperature (C) are shown.
(TIF)
