# Supplementary material for: Exploring risk factors for insect borer attack in Georgia’s (USA) urban landscapes
Source: PLoS One. 2024 Feb 26;19(2):e0299368. doi: 10.1371/journal.pone.0299368 (PMC10896510; doi:10.1371/journal.pone.0299368)
Supplement: S1 Table — Details of the location and characteristics of selected sites in urban areas in Atlanta, GA (A) and Augusta, GA (B) were surveyed from July to August 2021 and 2022. (DOCX) [file pone.0299368.s001.docx]

**S1 Table. Details of location and characteristics of selected sites in urban areas in Atlanta and Augusta surveyed from July to August in 2021 and 2022.**

| **County** | **Site Type** | **Trees** | **Site Age (Years)** | **Site Area (m²)** | **Surrounding Areas** | **Host Trees in Site** |
| --- | --- | --- | --- | --- | --- | --- |
| **2021** |  |  |  |  |  |  |
| Clayton | Parking Lot | 32 | 19 | 21823 | Buildings, Road | *P. chinensis, Zelkova serrata, A. rubrum* |
| Clayton | Parking Lot | 24 | 19 | 6252 | Buildings, Open Area, Road | *U. parvifolia* |
| Cobb | Parking Lot | 25 | 9 | 7866 | Buildings, Road | *U. parvifolia, A. rubrum* |
| Columbia | Greenspace | 17 | 22 | NA | Buildings, Forest, Open Area, Road | *P. calleryana, Tilia cordata* |
| Columbia | Parking Lot | 56 | 11 | 5247 | Forest, Road | *Q. texana, Quercus lyrata, A. rubrum, A. buergerianum, Q. phellos* |
| Columbia | Parking Lot | 48 | 16 | 33591 | Buildings, Forest, Road | *Q. rubra, Q. texana, A. rubrum, Q. phellos* |
| Columbia | Parking Lot | 34 | 14 | 17414 | Buildings, Forest, Road | *Q. texana, A. rubrum, Q. phellos* |
| Columbia | Parking Lot | 32 | 3 | 9979 | Buildings, Road | *U. parvifolia, Q. texana, A. buergerianum* |
| Columbia | Parking Lot | 26 | 4 | 3646 | Buildings, Forest, Road | *Ostrya virginiana, A. saccharum, F. americana* |
| Columbia | Parking Lot | 25 | 6 | 11298 | Buildings, Road | *Carpinus caroliniana, P. chinensis, Quercus lyrata, Qrubra* |
| Columbia | Parking Lot | 20 | 14 | 3641 | Forest, Open Area, Road | *A. rubrum, A. buergerianum, Quercus alba* |
| Columbia | Parking Lot | 20 | 15 | 13937 | Buildings, Forest, Road | *U. parvifolia, A. rubrum, Q. phellos* |
| Columbia | Parking Lot | 14 | 11 | 5126 | Buildings, Road | *U. parvifolia, A. rubrum, Q. phellos* |
| Columbia | Parking Lot | 13 | 7 | 6691 | Buildings, Forest, Road | *P. chinensis, A. buergerianum, Salix babylonica* |
| Columbia | Parking Lot | 12 | 12 | 4325 | Buildings, Forest, Road | *A. rubrum* |
| Columbia | Parking Lot | 8 | 11 | 3815 | Buildings, Forest, Open Area, Road | *Q. texana, A. buergerianum, Quercus alba* |
| Coweta | Parking Lot | 69 | 10 | 17894 | Buildings, Road | *Carpinus caroliniana, P. calleryana, P. chinensis, U. parvifolia, A. rubrum, P.* x *yedoensis* |
| Coweta | Parking Lot | 42 | 6 | 14058 | Buildings, Road | *G. biloba, U. parvifolia, A. rubrum* |
| Fayette | Greenspace | 20 | 3 | NA | Buildings, Road | *U. parvifolia, A. buergerianum* |
| Fulton | Parking Lot | 51 | 5 | 14997 | Buildings, Road | *U. parvifolia, Q. texana* |
| Fulton | Parking Lot | 33 | 5 | 5627 | Buildings, Road | *F. grandifolia, U. parvifolia, Q. palustris, A. buergerianum* |
| Fulton | Parking Lot | 24 | 22 | 7492 | Buildings, Forest, Open Area, Road | *A. ginnala, U. parvifolia, Q. texana, A. rubrum* |
| Gwinnett | Parking Lot | 41 | 8 | 19748 | Buildings, Road | *P. chinensis, Q. phellos* |
| Gwinnett | Parking Lot | 30 | 4 | 6943 | Buildings, Road | *U. parvifolia, Q. rubra, Q. palustris, Quercus coccinea, A. buergerianum* |
| Henry | Parking Lot | 33 | 6 | 13778 | Buildings, Road | *G. biloba, U. parvifolia, Q. texana, A. rubrum* |
| Henry | Parking Lot | 31 | 6 | 8952 | Buildings, Forest, Road | *U. americana, Q. palustris, A. rubrum, Acer saccharinum, Q. phellos* |
| Richmond | Parking Lot | 38 | 10 | 30956 | Buildings, Open Area, Road | *F. grandifolia, P. chinensis, Q. texana* |
| Richmond | Parking Lot | 23 | 14 | 6287 | Buildings, Road | *F. grandifolia, U. americana* |
| Rockdale | Parking Lot | 14 | 9 | 10205 | Buildings, Road | *Q. palustris, A. rubrum, Q. phellos* |
| Spalding | Parking Lot | 22 | 11 | 7833 | Buildings, Open Area, Road | *P.* x *yedoensis* |
| **2022** |  |  |  |  |  |  |
| Cherokee | Green Space | 16 | 7 | NA | Forest, Open Area, Road | *Nyssa sylvatica, G. biloba, Betula nigra, A. saccharum* |
| Cherokee | Parking Lot | 39 | 7 | 20051 | Buildings, Forest, Open Area, Road | *P. chinensis, Q. shumardii* |
| Cherokee | Parking Lot | 16 | 8 | 5681 | Buildings, Road | *A. saccharum* |
| Cherokee | Parking Lot | 11 | 15 | 10982 | Buildings, Road | *U. parvifolia, A. saccharum* |
| Cobb | Parking Lot | 84 | 15 | 36527 | Buildings, Forest, Road | *Zelkova serrata, Q. palustris, A. rubrum, Betula nigra, Q. shumardii, Q. phellos* |
| Cobb | Parking Lot | 30 | 6 | 16479 | Buildings, Road | *U. parvifolia, Q. shumardii* |
| Cobb | Parking Lot | 15 | 11 | 4843 | Buildings, Road | *A. rubrum, Liquidambar styraciflua, A. buergerianum, Q. phellos* |
| Cobb | Parking Lot | 12 | 11 | 9669 | Buildings, Road | *Zelkova serrata, U. parvifolia, Q. phellos, P.* x *yedoensis* |
| Coweta | Parking Lot | 20 | 17 | 14210 | Buildings, Road | *U. parvifolia, A. rubrum* |
| DeKalb | Park/ Green Space | 15 | 11 | NA | Buildings, Forest, Open Area, Road | *Q. palustris, A. rubrum, Q. shumardii, A. saccharum* |
| Douglas | Parking Lot | 17 | 8 | 16695 | Buildings, Forest, Road | *P. chinensis, A. buergerianum* |
| Fayette | Parking Lot | 19 | 13 | 13749 | Buildings, Forest, Road | *A. ginnala, U. parvifolia, A. rubrum, Q. phellos* |
| Fayette | Parking Lot | 18 | 16 | 21815 | Buildings, Forest, Road | *A. rubrum, Q. shumardii* |
| Fayette | Parking Lot | 12 | 6 | 8990 | Buildings, Road | *U. parvifolia* |
| Fayette | Parking Lot | 10 | 6 | 1520 | Buildings, Forest, Road | *Q. texana, A. rubrum, P.* x *yedoensis* |
| Forsyth | Parking Lot | 79 | 7 | 32255 | Buildings, Forest, Open Area, Road | *Q. texana, Q. palustris, A. rubrum, Q. shumardii, Q. saccharinum, A. buergerianium, Q. phellos* |
| Fulton | Parking Lot | 20 | 4 | 11541 | Buildings, Road | *P. chinensis, Q. phellos* |
| Gwinnett | Parking Lot | 18 | 16 | 10630 | Buildings, Forest, Road | *Q. texana, Q. palustris, A. rubrum, Q. shumardii* |
| Gwinnett | Parking Lot | 11 | 7 | 4297 | Buildings, Road | *U. parvifolia, A. rubrum* |
| Henry | Parking Lot | 12 | 12 | 10642 | Buildings, Road | *A. rubrum, Q. shumardii, P.* x *yedoensis* |
