# Supplementary material for: Exploring risk factors for insect borer attack in Georgia’s (USA) urban landscapes
Source: PLoS One. 2024 Feb 26;19(2):e0299368. doi: 10.1371/journal.pone.0299368 (PMC10896510; doi:10.1371/journal.pone.0299368)
Supplement: S2 Table — (DOCX) [file pone.0299368.s002.docx]

**S2 Table. AIC and BIC scores for the comparison of nonlinear exponential regression and zero inflated negative binomial models.**

|  | **Flatheaded Borer** | | **Bark Beetle** | | **Borers Overall** | |
| --- | --- | --- | --- | --- | --- | --- |
|  | **AIC** | **BIC** | **AIC** | **BIC** | **AIC** | **BIC** |
| **Nonlinear Exponential Regression** | 961.738 | 996.6698 | 437.5462 | 472.478 | 1054.171 | 1089.103 |
| **Zero Inflated Negative Binomial** | 669.8169 | 735.314 | 262.4037 | 327.9008 | 813.153 | 878.6501 |
