# Supplementary material for: Exploring risk factors for insect borer attack in Georgia’s (USA) urban landscapes
Source: PLoS One. 2024 Feb 26;19(2):e0299368. doi: 10.1371/journal.pone.0299368 (PMC10896510; doi:10.1371/journal.pone.0299368)
Supplement: S3 Table — Number of flatheaded borer (A), bark beetle (B), and total borer (C) holes by tree species. (DOCX) [file pone.0299368.s003.docx]

**S3 Table. Number of flatheaded borer (A), bark beetle (B), and total borer (C) holes by tree species.**

| **Tree Species** | **Total *n*** | ***n* with flatheaded borer attack** | ***n* with bark beetle attack** | ***n* with any borer attack** |
| --- | --- | --- | --- | --- |
| ***A. buergerianum*** | 20 | 2 | 1 | 3 |
| ***A. ginnala*** | 14 | 1 | 1 | 1 |
| ***A. rubrum*** | 240 | 74 | 8 | 76 |
| ***A. saccharum*** | 32 | 0 | 0 | 0 |
| ***F. grandifolia*** | 26 | 1 | 0 | 1 |
| ***G. biloba*** | 18 | 1 | 1 | 2 |
| ***P. chinensis*** | 103 | 0 | 1 | 1 |
| ***P.* x *yedoensis*** | 37 | 7 | 8 | 15 |
| ***P. calleryana*** | 20 | 0 | 0 | 0 |
| ***Q. palustris*** | 34 | 0 | 0 | 0 |
| ***Q. phellos*** | 128 | 6 | 0 | 6 |
| ***Q. shumardii*** | 81 | 0 | 0 | 0 |
| ***Q. texana*** | 135 | 7 | 2 | 9 |
| ***U. americana*** | 26 | 0 | 1 | 1 |
| ***U. parvifolia*** | 274 | 0 | 3 | 3 |
