# Supplementary figures and images for: Exploring risk factors for insect borer attack in Georgia’s (USA) urban landscapes
Source: PLoS One. 2024 Feb 26;19(2):e0299368. doi: 10.1371/journal.pone.0299368 (PMC10896510; doi:10.1371/journal.pone.0299368)

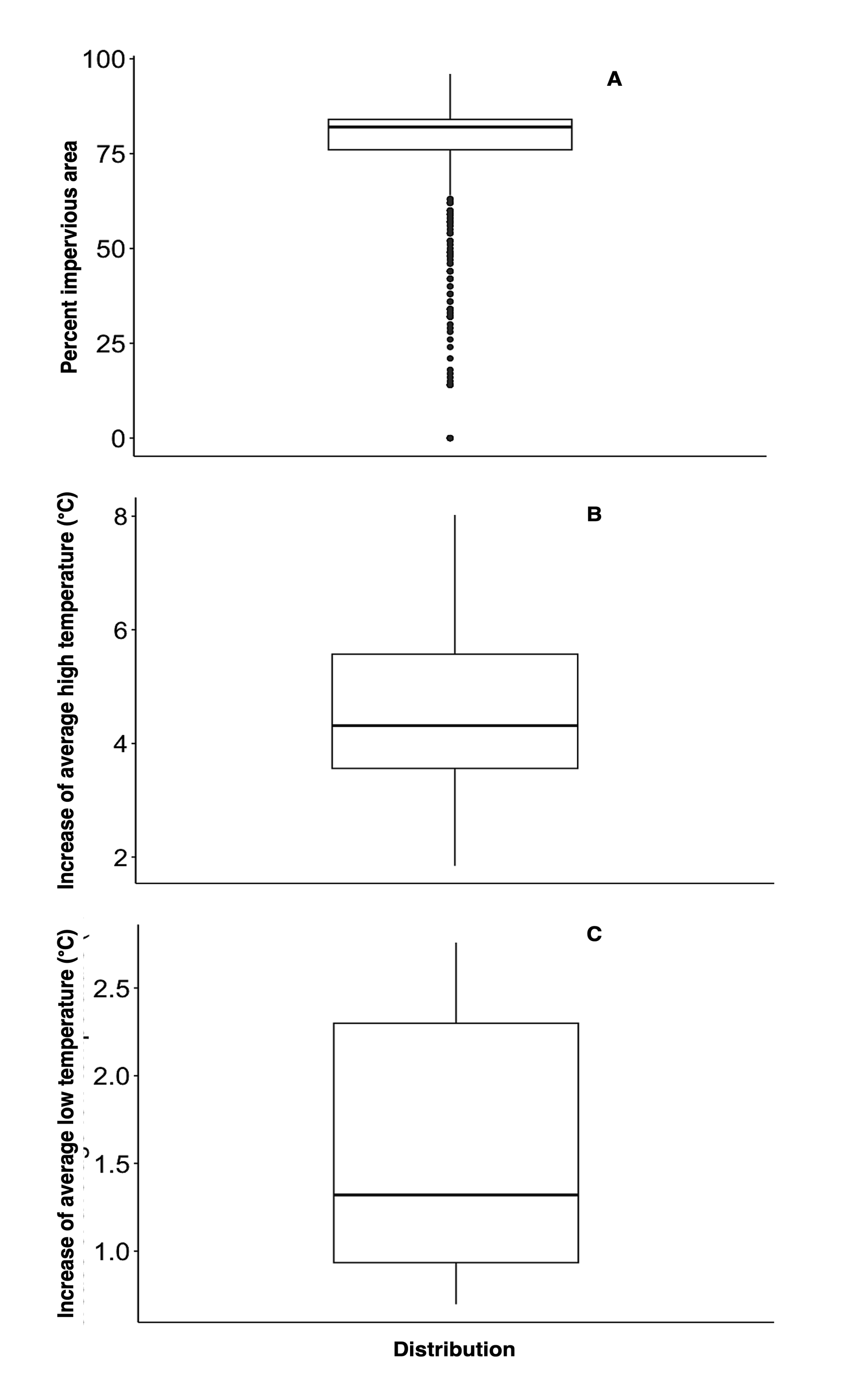

Supplement: S1 Fig — Distributions of percent impervious area (A), increase of average higher temperature (B), and increase of average low temperature (C) are shown. (TIF) [file pone.0299368.s004.tif]
